# Amalgam as a Filling Material

**Published:** 1905-12-15

**Authors:** Geroge Zederbaum


### AMALGAM AS A FILLING MATERIAL.

BY GEORGE ZEDERBAUM, D.D.S.

Read before the Michigan State Dental Association, July 12th, 1905.

Amalgam, as everything else in the way of filling materials, has its good and its bad qualities; has its friends and its enemies.

It is not my purpose to uphold the operator who uses lots of amalgam, and who, when it comes to anterior teeth, always finds them too soft and not capable of being filled with gold, but uses cement periodically until finally the tooth must be cut off and crowned; nor is it my aim to side in with the chronic gold worker, who, perhaps does not do any amalgam work, but spends all his energy and taxes all his and his patient's strength by malleting hours on a disto-occlusal cavity in a second upper molar; nor is it my desire to reflect upon the porcelain worker, who is so enthused with his material, which is centainly ideal in many ways, that he uses it to the total exclusion of all others. Personally I am neither overenthusiastic nor extremely conservative over amalgam, and the object of this paper is to outline with absolute fairness, the value of amalgam as compared with gold and with porcelain.

A short history of amalgam ought to be of interest, inasmuch as no other filling material has caused so much controversy as has amalgam. It has been in use since 1826, and was brought to the United States by two Frenchmen in 1830. It had to fight its way from the first, and if we are to judge by the output of that material by all the dental houses to-day, we can truthfully say that amalgam has won its battle and has come to stay, inasmuch as this great supply is certainly caused by a correspondingly great demand.

Now let us see what some of the most eminent of our profession have to say relative to amalgam:

Dr. G. V. Black says: "Amalgam produced by the best makers possesses many good qualities, yet is inferior to gold in all."

Dr. Jonathan Taft says: "Of all the metals that have been used for filling teeth, gold possesses more of the requisite properties than any other; sufficiently so for all practical purposes."

Dr. G. A. Bennett's article in the American system of dentistry reads: "Gold having the largest number of required attributes has always been and still is the most useful metal for filling teeth. Perhaps the most general rule can be given for amalgam is this—keep it out of sight."

Dr. J. S. Marshall says: "Amalgam is generally used under those conditions and circumstances in which a permanent filling is desired, but in which gold is inadmissible either from the inaccessibility of the cavity, the frail condition of the tooth, the physical state of the patient, or the inability of the individual to pay for the more expensive services."

To sum it all up, none of these gentlemen are over-enthusiastic in their praises of amalgam, and still we use it daily, and its output, as I said before, is steadily on the increase. Surely it is not because of its bad qualities that it is so lavishly pressed upon the public. It must have some redeeming features as well!

Some of the good points of amalgam may be summarized as follows:

Apparent adaptability to walls of cavities.

Resistance to attrition.

Edge strength.

Conductivity of thermal changes not as high as that of gold.

Working qualities and ease of manipulation.

Less time in placing.

Less expenditure of force is required.

If the filling is properly made, amalgam we are using now-a-days, will not discolor the teeth.

Slight cost of material.

Amalgam is of especial value in that class of cavities in which the cervical margin lies at some distance beneath the border of the gum; also in large disto-occlusal cavities of second and third molars, which by reason of their location, their size and frail walls, or the physical condition of the patient, gold filling or gold or porcelain inlays could not be satisfactorily placed.

On the other hand there are some bad qualities of amalgam, as for instance:

It is not entirely indestructible in the fluids of the mouth, but oxidizes or sulphurets.

Resistance to the force of mastication is not as good as that of gold.

Color is bad; inadmissible for that reason for anterior teeth.

Much careful study and experience are required to work it well. (Neutral point.)

Cavities to be filled with amalgam sucessfully must always have four surrounding walls.

Ordinarily, perfection of adaptation is less certain than in case of gold.

There is danger of occasional mercurial necrosis from too many amalgam fillings in one mouth. (See Brooklyn Med. Journal, Dec. 1898.)

Owing to cheapness, too many of us handle it carelessly.

Lactic acid producing germs are not excluded by amalgam.

Too many inferior alloys on the market.

In comparing amalgam with gold fillings, I believe that we put in entirely too many amalgam fillings, where gold ones would give far better satisfaction. It is true that gold requires more skillful operating from beginning the preparation of the cavity to the final polishing of the filling, but there certainly are conditions, as before mentioned, where amalgam would be of better permanency than gold. Let me say right here that one point in the operative procedure is decidedly neglected by the majority of practitioners and that is *rubber dam.* Many amalgam fillings would be perfect to-day, had they been put in as they should have been. To willfully neglect the application of the rubber dam is unpardonable. I distinctly remember how fixed on this point was Dr. Black, when he insisted on rubber dam for even shallow occlusal pit cavities. This vulnerable point certainly should not be passed over lightly, especially by those who even do not employ the compressed air.

As to the life of amalgam fillings I do not know what to say. Much depends on conditions I presume. I know I have two very large amalgam fillings in my mouth since 1899 and that to-day, without any extraordinary care, they are there as bright and with as smooth margins as you could wish for. When properly placed and under at all favorable conditions amalgam fillings serve well.

Before we compare the value of amalgam with a gold inlay, let us first compare the large gold filling with the gold inlay.

If the insertion of a good gold filling requires great skill and manipulative ability then a good gold inlay requires still more dexterity and sense of touch. The making and the inserting of the gold inlay is decidedly more pleasant and less tedious operation than a large gold filling. We can place a gold inlay with a fair degree of assurance of permanency in teeth of young patients, giving them all the benefits of cements without their disadvantages. Then again, in cases of devitalized posterior teeth where as a natural sequence we have to deal with frail walls, we can protect these walls by inlays better than with any gold filling that can be placed in them.

From these few points mentioned (and there are many more), we can see that a gold inlay is a good thing, and in many cases better than a gold filling. The main drawbacks, as I see them, are: the cutting away of so much sound tissue to enable one to perfectly draw the burnished matrix occlusally; contraction of solder in cooling, few can master this contraction, and the cement trouble, which is likewise a weakness in the porcelain inlay work. As repeatedly shown by experiments and clinical experience we learned the fact that we as yet lack suitable material with which we can hermetically secure our inlays. Cement washes out, in many cases, especially where it has been allowed to cover the cavo-surface of the enamel. This dissolves with greater or less rapidity, all depending on some peculiar idiosyncrasy of the patient so that scarcely any time elapses before the inlay has to be re-cemented. However I regard gold inlay superior to the gold filling, and as the gold filling has been universally allowed supremacy over the amalgam filling—natural deduction is—gold inlay is better than amalgam filling, all conditions being equal.

Now then we come to the comparatively value of amalgam with the *porcelain* inlay. I am not going into relative merits and demerits of porcelain inlay versus amalgam filling. Each has its respective place in the mouth and these two certainly ought not to conflict. Amalgam has its place for the posterior teeth, while the field of porcelain inlay, with occasional exception, should be confined to the anterior teeth, and only to such of these where there is or there can be made sufficient space, and where the stress is not great and sufficient retention can be secured in simple buccal cavities where we are perfectly safe as far as the stress goes.

While the porcelain is by far more artistic than either the gold inlay or the amalgam filling, and while it is also more compatible with tooth structure, it also has its faults and its limitations. I will enumerate just a few, such as chipping which frequently occurs (no edge strength), for, even from a good joint cement will sometimes wash out and crushing strain from the opposing tooth will chip the unsupported porcelain, thus inviting accumulations of food and further decay and further washing out of cement, at times entirely loosening the inlay. Many porcelain inlays discolor at the margins, which is also due to partial dissolving out of the cement. We can not place porcelain inlays in posterior teeth with as great certainty of success as we can amalgam. Further, we know that dentine is permeated with lactic acid fermentative process in advance of decay and for that reason we are never positive that the filled cavity will not have to be re-opened and tooth treated. Imagine the removal of a mechanically, well placed porcelain inlay from a very sore tooth. Patient would rather lose the tooth than be obliged to sit while you pry, drill and otherwise try to dislodge such an inlay. On the other hand, amalgam could be readily drilled out with a sharp drill in a jiffy, which might be an advantage in this case, of amalgam over the porcelain inlay.

In closing I wish to express my belief that, although amalgam is a cheaper material, requiring less manipulative skill than either the gold or porcelain, that is no reason why its insertion should be less carefully or less conscientiously performed.

I do not pray for more amalgam fillings, no, we have too many of them already, but I certainly do plead for better cavity preparation, better placed and better finished amalgam fillings.

---

